# Platypnoea-orthodeoxia syndrome, an underdiagnosed cause of hypoxaemia: four cases and the possible underlying mechanisms

**DOI:** 10.1007/s12471-015-0714-5

**Published:** 2015-07-14

**Authors:** M. Nassif, H. Lu, T.C. Konings, B.J. Bouma, A. Vonk Noordegraaf, B. Straver, N.A. Blom, S.A. Clur, A.P.C.M. Backx, M. Groenink, S.M. Boekholdt, D.R. Koolbergen, M.G. Hazekamp, B.J.M. Mulder, R.J. de Winter

**Affiliations:** 1 Department of Cardiology, Cardiac Catheterization Laboratory, Academic Medical Center—University of Amsterdam, PO Box 22660, 1100 DD Amsterdam, The Netherlands; 2 Department of Cardiology, VU University Medical Center, Amsterdam, The Netherlands; 3 Department of Pulmonary Medicine, VU University Medical Center, Amsterdam, The Netherlands; 4 Department of Pediatric Cardiology, Academic Medical Center, Amsterdam, The Netherlands; 5 Department of Pediatric Cardiology, Leiden University Medical Center, Leiden, The Netherlands; 6 Department of Cardiothoracic Surgery, Academic Medical Center, Amsterdam, The Netherlands; 7 Department of Pediatric Cardiac Surgery, Leiden University Medical Center, Leiden, The Netherlands; 8 Interuniversity Cardiology Institute of the Netherlands (ICIN), Utrecht, The Netherlands

**Keywords:** Platypnoea-orthodeoxia, Dyspnoea, Foramen ovale, patent, Atrial septum, Mechanism

## Abstract

Cardiac platypnoea-orthodeoxia syndrome (POS) is a position-dependent condition of dyspnoea and hypoxaemia due to right-to-left shunting. It often remains unrecognised in clinical practice, possibly because of its complex underlying pathophysiology. We present four consecutive patients with POS and patent foramen ovale (PFO) who underwent a successful percutaneous PFO closure, describe the mechanism of their POS and provide a review of the literature.

## Introduction

Platypnoea-orthodeoxia syndrome (POS) is an uncommon clinical condition, characterised by position-dependent dyspnoea and oxygen desaturation in an upright position which resolves by lying supine. In addition to cardiac defects, pulmonary and abdominal defects have also been reported in association with POS (Tables 1 and 2) [1]. POS was first described in a patient with an atrial septal defect (ASD); however, cardiac POS has subsequently been more often observed in patients with a patent foramen ovale (PFO) [2]. What seems essential for cardiac POS to exist are both an anatomical communication between the right and left atrium as well as a structural component that redirects shunt flow causing right-to-left shunting in the upright position [3]. Usually this structural component is a deformation of the right atrium or of the inter-atrial septum. POS is primarily characterised by normal right atrial and pulmonary artery pressures contrary to Eisenmenger’s syndrome [4]. The exact mechanism of position-dependent change in shunting, including the gravitational forces on the cardiac structures involved, remains elusive. The final common pathway appears to be a position-dependent opening of the PFO with right-to-left shunting. We present four cases of POS treated at the Centre for Congenital Heart Disease Amsterdam Leiden (CAHAL), and discuss the pathophysiological mechanism of POS and its diagnostic and therapeutic pitfalls.

**Table 1:** Conditions associated with cardiac POS

Group A: anatomic preferential blood flow across the inter-atrial communication	Group B: transient reversal of left-to right pressure gradient
Congenital abnormality	Pulmonary
Absent superior vena cava	Chronic obstructive pulmonary disease
Ascending aortic aneurysm	Pneumonectomy
Atrial septal aneurysm	Pulmonary embolism
Ebstein’s anomaly	Pulmonary hypertension
Partial anomalous venous return	Cardiac
Persistent left superior vena cava	Constrictive pericarditis
Prominent Eustachian valve	Pericardial adipose deposition
Transposition of the great vessels	Pericardial effusion
Unroofed coronary sinus	
Post-surgical repair	
Aortic valve replacement	
Ascending aorta repair	
Atrial switch procedure	
Fontan procedure	
Tumours	
Cardiac cyst/mass	
Lipomatous hypertrophy of the inter-atrial septum	
Other	
Eosinophilic endomyocardial disease	
Hepatic cyst	
Tortuous ascending aorta	
Tricuspid regurgitation or stenosis	

Diseases associated with POS in case of inter-atrial communication. Conditions in group A cause POS through anatomical distortion of the right atrium (e.g. congenital abnormality, post-surgical repair, cardiac or hepatic cyst) or the atrial septum (e.g. a thin, floppy septum primum in lipomatous hypertrophy of the inter-atrial septum), or by disposition of the right ventricle (e.g. an inflamed, thick-walled ventricle due to eosinophilic endomyocardial disease) leading to preferential flow through the inter-atrial septum. In group B the pulmonary and cardiac conditions cause POS in the presence of a right-to-left pressure gradient due to pulmonary vascular resistance and compression of the right ventricular inflow tract respectively. Adapted from Knapper et al. [1].

**Table 2:** Non-cardiac conditions associated with platypnoea-orthodeoxia syndrome

Pulmonary	Abdominal	Other
Acute respiratory distress syndrome	Paralytic ileus	Chest wall trauma
Chronic obstructive pulmonary disease	Hepatopulmonary syndrome	Diabetic autonomic neuropathy
Cryptogenic fibrosing alveolitis	Alcoholic liver cirrhosis	Organophosphate poisoning
Fat embolism	Autoimmune hepatitits	Paraoesophageal hernia repair
Hemidiaphragmatic dysfunction	Hepatitis A	Parkinson’s disease
Pleural effusion	Noncirrhotic portal hypertension	Vertebral fractures
Pneumocystis and CMV pneumonia	Schistosomiasis	Kyphoscoliosis
Pneumonectomy		
Pulmonary arteriovenous malformations		
Pulmonary embolism		
Radiation-induced bronchial stenosis		
Traumatic bronchial rupture		

Non-cardiac associations with POS include pulmonary conditions which cause pulmonary vascular shunting (e.g. arteriovenous malformation), ventilation-perfusion mismatching (e.g. pleural effusion, pneumonia, bronchial stenosis) or an anatomic distortion of the right atrium or ventricle (e.g. hemidiaphragmatic dysfunction). Hepatopulmonary syndrome causes POS through a high alveolar-arterial gradient (e.g. in portal hypertension, hepatitis). Orthostatic hypotension as a symptom of autonomic dysfunction (e.g. Parkinson, diabetic autonomic neuropathy, organophosphate poisoning) causes POS through an increase in orthostatic alveolar dead space and subsequent ventilation-perfusion mismatching. *CMV* Cytomegalovirus.

## Case 1

**Fig. 1:**
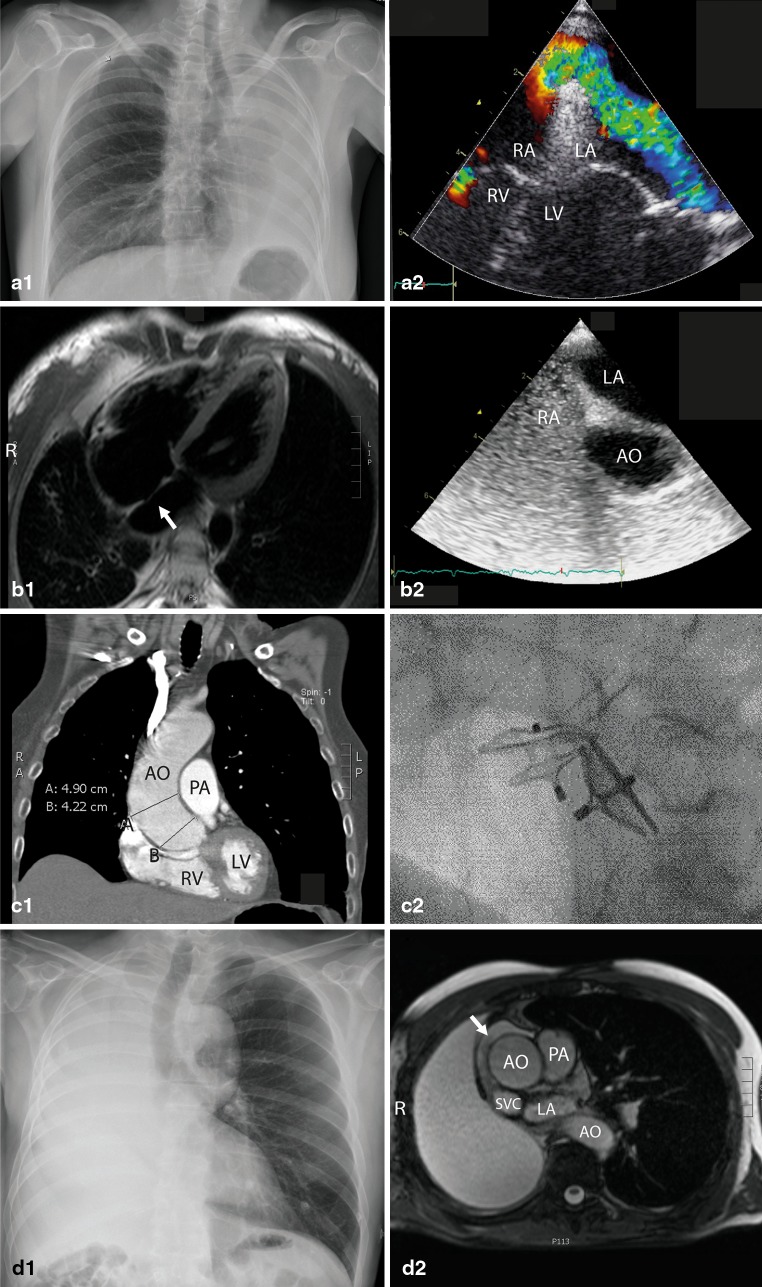
Different imaging modalities of case 1(**a**), 2 (**b**), 3(**c**) and 4 (**d**), respectively, for the purpose of diagnosing POS by PFO. **a1** Chest X-ray showing the heart position against the left thoracic wall. **a2** Four chamber view of a Doppler TEE with a right-to-left shunt by PFO. **b1** CMR showing malposition of the right thoracic wall resulting in a heart shift to the right and presence of PFO (*arrow*). **b2** Short axis basal view of a Bubble contrast TEE showing no resting right-to-left shunting over the PFO. **c1** CT angiography showing the dilated ascending aorta (*A*) and aorta root (*B*). **c2** Periprocedural angiographic image of both Amplatzer devices. **d1** Chest X-ray showing a tracheal shift to the left after pneumonectomy. **d2** CMR shows a right hemithorax filled with pleural effusion and a compressed right atrium (*arrow*). *RA* right atrium, *LA* left atrium, *RV* right ventricle, *LV* left ventricle, *R* right, *AO* aorta, *PA* pulmonary artery, *SVC* superior vena cava

A 46-year-old woman was referred to our hospital for percutaneous PFO closure, after having experienced symptoms of dyspnoea in the upright and left supine position for several months. She had a history of chronic obstructive pulmonary disease GOLD II and had undergone left pneumonectomy 6 months earlier, to remove an adenocystic carcinoma. In the weeks, thereafter, she developed progressive and position-dependent shortness of breath. Physical examination revealed a blood pressure of 100/65 mmHg with a resting heart rate of 122 beats/min. She had central cyanosis, with a transcutaneous oxygen saturation of 91 %, while breathing 1.5 L oxygen as measured by pulse oximetry. Her heart sounds were shifted to the left on auscultation. Chest X-ray (Fig. 1a1) and computed tomography (CT) scan of the thorax showed a midline shift of the right lung with a consequent position of the heart to the left thoracic wall. No evidence was found of cancer recurrence or other pulmonary causes such as pleural effusion or atelectasis. Echocardiographic images revealed a PFO (Fig. 1a2) and a right-to-left shunt fraction measured 20 % when breathing 100 % oxygen in an upright position. At this point the patient was severely incapacitated; she could only lie on her right side, which had resulted in severe muscular atrophy. POS was finally diagnosed 6 months after the onset of her symptoms. Cardiac catheterisation was performed, which showed a normal right atrial and pulmonary artery pressure, and pulse oximetric saturations of 90 % and 83 % in right and left supine positions, respectively. The PFO was successfully closed with a 25 mm Amplatzer PFO Occluder (St. Jude, Minneapolis, USA) after which the arterial saturation improved immediately to 97 % in the upright position. Her dyspnoea was resolved but an extensive rehabilitation course followed. She was able to gradually resume her daily routine and her exercise capacity improved significantly. No complications have been reported in 3 years of clinical follow-up. On her last transthoracic echocardiogram (TTE), the estimated systolic pulmonary artery pressure was 28 mmHg and no residual shunt was seen over the closed PFO.

## Case 2

A 36-year-old woman with a history of right mid and lower lobe resection due to a carcinoid in the right main bronchus 3 years earlier was referred to our hospital. She complained of progressive pain and dyspnoea since her operation, which was most prominent during exercise and when lying in the right supine position. Before the bilobectomy she had participated in competitive long distance running, but now the referring pulmonologist noted that she desaturated from 96 to 87 % with severe dyspnoea after performing only10 squats. Previously, a bronchoscopy revealed an open but narrow bronchus to the right upper lobe. This narrowing worsened in the right supine position and was therefore thought to be the cause of her dyspnoea in the right supine position. One year later, cardiac magnetic resonance (CMR) imaging and resting transoesophageal echocardiography (TEE) showed a PFO without a right-to-left shunt as measured in normal supine position so no POS was suspected (Fig. 1b1–2). None of the previous diagnostic tests could explain the coexistent position-dependent and exercise-induced dyspnoea. A cardiopulmonary exercise test showed an early arterial partial CO_2_ pressure (PaCO_2_) increase during exercise. Also, an increase of end-tidal O_2_ pressure and decreased end-tidal CO_2_ pressure were seen at an acute load of 150 W, with a desaturation from 96 to 93 % seen on pulse oximetry. Both her pulmonologist and cardiologist agreed that her symptoms had to be attributed to a mechanism involving her PFO. Firstly, the exercise-induced right-to-left shunt itself, and secondly, the consequently triggered ventilatory impairment related to the loss of alveolar tissue after pulmonary resection preventing adequate compensatory hyperventilation to normalise the PaCO_2_. Subsequently, the PFO was closed successfully with a Premere 25 mm device (St. Jude, Minneapolis, USA). She underwent extensive cardiac rehabilitation and on her latest outpatient visit, after 4 years of follow-up, no complications were found. She reported having run a marathon again, despite her unchanged ventilatory impairment. Her final TTE showed a normal pulmonary artery pressure and no residual shunt.

## Case 3

A 73-year-old woman presented to our emergency department with acute dyspnoea and a severe hypoxaemia with a saturation of 63 % measured by pulse oximetry in the upright position. She had a history of frequent recurrent pulmonary embolism due to essential thrombocytosis; however, a CT scan of the thorax excluded recurrent pulmonary embolism as a cause of dyspnoea this time. A TTE was performed to exclude pulmonary hypertension. Surprisingly, the echocardiogram in the left supine position revealed a PFO and a mildly elevated systolic pulmonary artery pressure of 36 mmHg. A subsequent TEE confirmed the existence of POS; pulse oximetric saturations dropped from 100 % and 82 % within 60 s when the patient changed from supine to upright position. Coincidentally, she was known with a stable aortic root and ascending aorta dilatation (diameters of 42 mm and 49 mm respectively, Fig. 1c1). The PFO was successfully closed with an Amplatzer 18 mm PFO Occluder. Due to a residual shunt on the procedural TEE, an additional 6 mm ASD Septal Occluder device was also implanted (Fig. 1c2) and the PFO was successfully closed. At one year follow-up she had no residual shunt and she had physically recovered from her former incapacitated condition.

## Case 4

A 61-year-old male patient was referred to our hospital’s congenital team for PFO closure due to POS. He had a history of pulmonary adenocarcinoma and had undergone a right pneumonectomy 6 months earlier. After the subsequent chemotherapy, he complained of progressive general deterioration and dyspnoea in the upright position. On presentation he was unable to walk more than five steps. Physical examination showed saturations of 97 % versus 90 % in the supine and upright position, respectively, measured by pulse oximetry with 3 L oxygen. Analysis by contrast echocardiography revealed a right-to-left shunt due to a PFO with a measured shunt fraction of 26 %. The chest X-ray showed a complete shift of the heart to the right (Fig. 1d1). CMR showed pleural effusion in the right hemithorax (Fig. 1d2) and no evidence was found for shunting in the supine position. During diagnostic heart catheterisation, systolic and diastolic pulmonary artery pressures were 33 mmHg and 16 mmHg, respectively, when lying supine, and 48 mmHg and 28 mmHg, respectively, in the upright position. Arterial blood gas during catheterisation provided oxygen saturations of 96 % and 85 % in supine and upright position. Due to the patient’s debilitated condition and severe hypoxaemia with 5 L oxygen, an urgent percutaneous PFO closure was performed with an 8 mm Amplatzer Septal Occluder. He recovered rapidly and was able to walk around the ward within 2 days with saturations of 97 % in both supine and upright positions, after having only been able to lie in a supine position for the previous 3 weeks of his hospital stay. A TTE before hospital discharge showed no residual shunt. His follow-up after 4 months was uneventful with a near-normal exercise capacity.

## Discussion

We describe four cases with platypnoea-orthodeoxia syndrome caused by the combination of patent foramen ovale and a pulmonary condition with loss of pulmonary volume. The mechanism by which POS develops in the absence of pulmonary hypertension is often unclear. Two explanations have been postulated to explain POS in the presence of an inter-atrial communication: (1) a position-dependent transient pressure gradient across the inter-atrial septum and (2) an anatomical preferential blood flow through the inter-atrial communication [4–6].

**Fig. 2:**
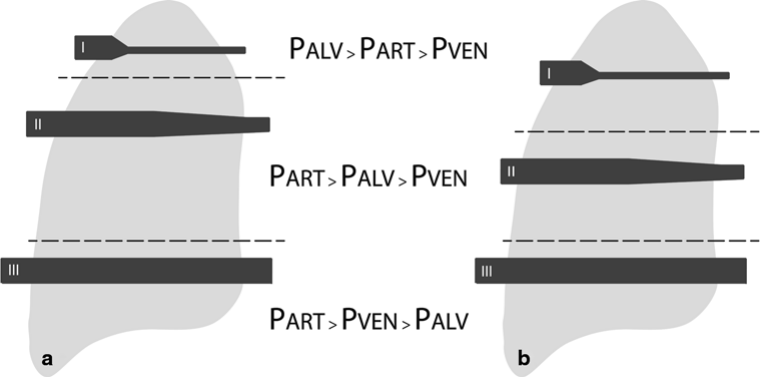
A physiological model of the pulmonary vasculature in the upright position in a normal lung (**a**) versus a lung post-pneumonectomy (**b**). Due to gravitation, in the upright position blood flow in the apex of the lung is physiologically prevented since alveolar pressure exceeds the pulmonary arteriolar pressure (pulmonary zone I phenomenon). A high pulmonary vascular resistance in the post-pneumonectomy situation causes an increase in right ventricular afterload. When right ventricular output reduces in the upright position, this afterload cannot be compensated. Consequently, pulmonary arteriolar pressure drops even more, causing a larger pulmonary zone I. *I* pulmonary zone I with restricted blood flow, *II* zone II with normal blood flow, *III* zone III with maximum blood flow; *Palv* alveolar pressure, *Part* arteriolar pressure, *Pven* venous pressure

**Fig. 3:**
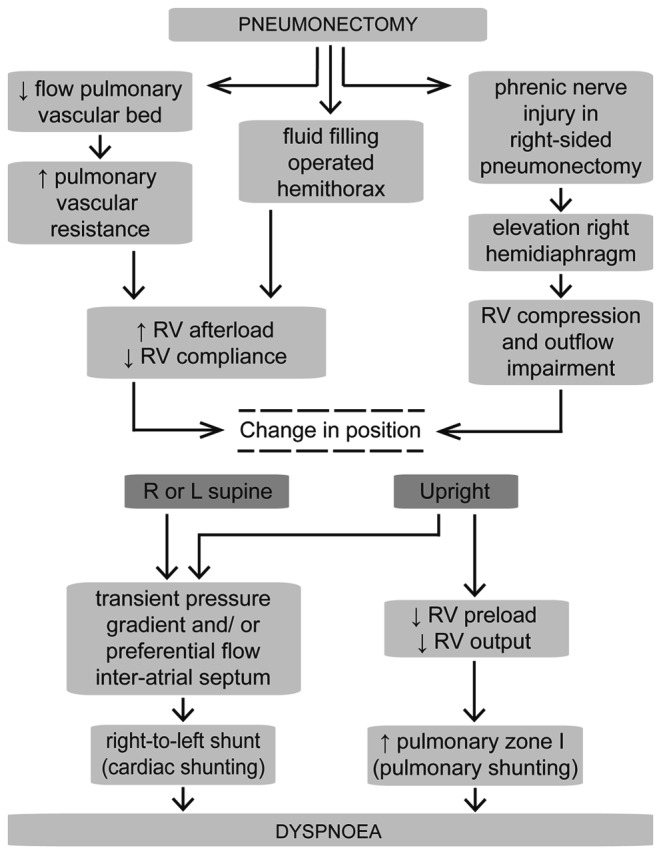
Schematic view of the haemodynamic mechanism explaining the position-dependent dyspnoea after pneumonectomy in case of an inter-atrial communication. Through several pathways pneumonectomy can result in dyspnoea by a position-dependent transient pressure gradient or an anatomical preferential flow across the inter-atrial septum. In the upright position gravity leads to additional pulmonary shunting. *RV* right ventricle, *R* right, *L* left

Case 1, 2 and 4 had undergone pulmonary resection, in one patient this was left-sided and in two patients right-sided, after which they started developing dyspnoea. According to the first explanation, after pneumonectomy the right ventricular afterload increases and compliance decreases due to the reduced pulmonary vascular bed (Fig. 1a1, b1, d1) and the subsequent increase in pulmonary vascular resistance. Right ventricular afterload is also increased by post-pneumonectomy fluid overload in the operated hemithorax [7, 8]. These haemodynamic changes cause a right-to-left shunt due to a transient pressure gradient since right atrial pressure increases. An upright position reduces right ventricular preload and cardiac output. Orthodeoxia might consequently develop due to an increase in the alveolar dead space known as ‘pulmonary zone I’ (Fig. 2), in which the pressure in alveoli exceeds the orthostatic decrease in pulmonary arteriolar pressures, causing an additional ventilation-perfusion mismatch [8]. Though not reported in our cases, a different cause of dyspnoea after lung surgery can be phrenic nerve injury, particularly after right-sided pneumonectomy [9, 10]. The elevated right hemidiaphragm causes right ventricular compression and subsequent outflow impairment, which in turn causes a transient pressure gradient resulting in the right-to-left shunt. In these particular cases, plication of the diaphragm is preferred over PFO closure to remove the underlying cause [9]. Figure 3 summarises the haemodynamic explanation in a schematic view.

The second explanation is the relocation of the atrial septum which facilitates preferential flow of caval venous blood into the opening of the atrial defect. Intrathoracic post-pneumonectomy changes also account for this positional change of the inter-atrial communication, along with the haemodynamic shift as described above. A spontaneous mediastinal relocation to the opposite side, which occurs approximately 3 weeks to 7 months after pneumonectomy, predisposes to stretching and distortion of the atrial septum as the inferior caval vein remains in place [11]. In the upright position additional stretching occurs due to gravity, which enlarges the inter-atrial communication and causes right-to-left shunting despite normal right atrial pressures [3, 4]. In a review of reported POS cases, Marini et al. [12] noted a significantly higher prevalence of POS in post-pneumonectomy patients who developed respiratory symptoms within 6 ± 7 months compared with those with a symptomless interval of < 1 month. Case 1, 2 and 4 indeed developed progressive dyspnoea within weeks to months after lung resection. The gradual synergic effect of atrial septal stretching along with a possible transient pressure gradient could explain the late onset of POS as seen in most reported cases [1, 6, 12].

Case 3 demonstrates POS caused by a PFO in a patient with elevated pulmonary artery pressure secondary to chronic pulmonary embolism. The haemodynamic mechanism involves elevated right atrial pressures due to relatively decreased right ventricular compliance, comparable with the post-pneumonectomy situation. Furthermore, in accordance with the preferential transseptal flow mechanism, she had a dilated aortic root and ascending aorta which in the upright position can horizontalise the atrial septum increasing caval venous flow through the defect, as well as compressing the right atrium to make the PFO more mobile and permeable for shunting [13–15].

Several important observations are worth mentioning. Firstly, in all presented cases POS was diagnosed several months after the onset of symptoms and clinical presentation. Beside the fact that clinicians are relatively unfamiliar with POS as compared with other causes of dyspnoea, its complicated underlying mechanism often requires different diagnostic imaging modalities which add to the delay in establishing a plausible diagnosis. Therefore clinicians must consider diagnosing POS when patients present with position-dependent dyspnoea, even before the common causes of dyspnoea have been excluded. Secondly, an immediate symptom relief is present as soon as the PFO is successfully closed, as confirmed in this case series. Clinical verification of the efficacy of PFO closure is indicated when symptom continuation implies a residual shunt.

In conclusion, diagnosing POS remains a challenge since clinicians must recognise the heterogeneous presentation in severely dyspnoeic patients. Position-dependent dyspnoea and desaturation should provide the clue. Initial assessment of POS can be made by observing the platypnoea, pulse oximetry and positional echocardiography. In some cases, POS can only be confirmed after observing the immediate symptom relief after PFO closure. Physicians should be reminded that a PFO, in combination with a pulmonary condition, can become symptomatic at older age as shown in this case series. In such cases, the debilitating dyspnoea can be successfully relieved by closure of the inter-atrial communication. Most importantly, clinical awareness is essential to recognise POS and prevent unnecessary delays.

### Funding

None.

### Conflict of interest

None declared.
